# Any Role for Microbiota in Cholangiocarcinoma? A Comprehensive Review

**DOI:** 10.3390/cells12030370

**Published:** 2023-01-19

**Authors:** Alessandra Elvevi, Alice Laffusa, Camilla Gallo, Pietro Invernizzi, Sara Massironi

**Affiliations:** 1 Gastroenterology Unit, Fondazione IRCCS San Gerardo dei Tintori, 20900 Monza, Italy; 2 Dipartimento di Medicina e Chirurgia, Università degli Studi Milano-Bicocca, 20900 Monza, Italy

**Keywords:** cholangiocarcinoma, microbiota, biliary tract cancer, gut-liver axis

## Abstract

Alterations in the human microbiota have been linked to carcinogenesis in several cancers. To date, few studies have addressed the role of the microbiota in cholangiocarcinoma (CCA). Our work aims to update the knowledge about the role of the microbiota in the CCA microenvironment, and to highlight possible novel insights for the development of new diagnostic, prognostic, or even therapeutic strategies. We thus conducted a review of the literature. In recent years, great progress has been made in understanding the pathogenesis, the clinical and histological behavior, and the molecular profile of CCA. Much evidence suggests that the bile microbiota plays an essential role in biliary diseases, including CCA. Some studies have demonstrated that alterations in the qualitative and quantitative composition of the intestinal commensal bacteria lead to overall cancer susceptibility through various pathways. Other studies suggest that the gut microbiota plays a role in the pathogenesis and/or progression of CCA. The clinical implications are far-reaching, and the role of the microbiota in the CCA microenvironment may lead to considering the exciting implications of implementing therapeutic strategies that target the microbiota-immune system axis.

## 1. Introduction

Cholangiocarcinoma (CCA) is a group of rare epithelial cell malignancies, arising from the cholangiocellular epithelium, which are mostly adenocarcinomas [1].

CCA has always been considered a rare disease, accounting for less than 1% of all human cancers and about 10–15% of all primary liver cancers. However, in recent decades, the overall incidence has increased worldwide [2]. It is usually diagnosed in the seventh decade of life, with a slight preponderance in men.

Some conditions are well-known risk factors for the development of CCA. Choledochal cysts (e.g., Caroli disease), inflammatory bowel disease (IBD), primary sclerosing cholangitis (PSC), and chronic liver disease are strongly associated with CCA incidence and progression, as well as liver flukes (e.g., *Clonorchis Sinensis* and *Opisthorcis Viverrini*), especially in some countries (e.g., Korea, Thailand) [3,4]. All these risk factors indistinctly lead to chronic inflammation of the biliary epithelium and biliary stasis, which represent common features that thus play a fundamental role in the pathogenesis of CCA [5]. The increasing rate of CCA has led researchers to analyze new and emerging risk factors. Therefore, in addition to the well-known risk factors listed above, other pathogenesis hypotheses have been proposed, mainly addressed to the gut-liver axis: impaired gut barrier function and altered gut microbiota composition, in fact, have been proven to favor the translocation of gut bacteria to the biliary tract, which contributes to the maintenance of bile duct inflammation [6,7]. However, little is known to date about the role of this gut-biliary axis in the carcinogenesis of CCA, and few studies have addressed the specific influence of the gut microbiota in CCA incidence and progression. Knowledge of the potential role of the microbiota in the microenvironment of this cancer may provide new insights for the development of novel diagnostic, prognostic, or even therapeutic strategies.

## 2. Classification and Epidemiology of CCA

CCA is divided into three subtypes according to its anatomical site of origin: intrahepatic (iCCA), perihilar (pCCA), and distal (dCCA) CCA. iCCAs arise above the second-order bile ducts, whereas pCCA and dCCA respectively arise above and below the confluence between the common bile duct and the cystic duct; pCCA and dCCA can also be grouped under the term “extrahepatic” (eCCA). In terms of frequency, iCCAs account for 10–20% of CCAs, while 50% are pCCA and 30–40% are eCCA [5,8].

The different classification of CCA during the past years may have influenced its correct estimation of prevalence and incidence. Notwithstanding the methodological problems in obtaining and processing data, the global incidence of CCA has increased worldwide in recent decades [5]. In the last decade, in fact, the intrahepatic CCA incidence rate increased rapidly by 109%, from 0.67 per 100,000 in 2007 to 1.40 per 100,000 in 2016 [2]. This trend can also be explained by improved diagnostic techniques (radiologic, endoscopic, and histologic) and increased physician awareness of this malignant neoplasm [2].

Interestingly, iCCA has shown a stable rate in recent years in countries where a decrease in alcohol-related chronic liver disease and cirrhosis has been observed. In contrast, the same form of CCA has shown an increasing rate in European and American countries where alcohol consumption, hepatitis C virus (HCV) infection, obesity, and metabolic-dysfunction-associated fatty liver disease (MAFLD) have increased [2,9].

## 3. Established, Emerging, and Controversial Risk Factors for CCA

The landscape of risk factors for CCA is complex and constantly evolving; indeed, in recent years, new risk factors have been recognized in addition to the known ones. Of the established risk factors, some are common to all types of CCA, such as chronic biliary diseases, while others are more specifically associated with certain subtypes that follow a specific geographic distribution [2].

### 3.1. Chronic Biliary Disease

Chronic biliary disorders represent well-known risk factors for all types of CCA, as chronic biliary inflammation and, eventually, biliary stasis are the pathophysiologic mechanisms.

One of the major causes of chronic biliary disease in the Western world is PSC, a rare idiopathic, immune-mediated chronic cholestatic liver disease characterized by inflammation of the intra- and extrahepatic biliary tree with progressive fibrosis, biliary strictures, and consequent progressive liver failure [10]. PSC is closely associated with IBD [11]. CCA is the most common PSC-related cause of death [12]. Previous studies have found up to 400-fold higher risk of CCA in PSC patients with concurrent IBD compared to the general population [12]. To date, there is no clear consensus on the best prevention and surveillance strategy for PSC patients, although regular imaging has been associated with improved survival [13]. IgG4-related disease, especially the biliary-involving phenotype (IRC), might mimic PSC; it is a systemic fibroinflammatory disease with tumor-like swelling of involved organs, a lymphoplasmacytic infiltrate rich in IgG4+ plasma cells, variable degrees of storiform fibrosis, obliterative phlebitis, and often elevated serum IgG4 concentration. The distinction between IRC and PSC is important as the cholangiographic changes of IRC may resolve completely upon corticosteroid treatment and IRC is not a pre-malignant condition [14].

A well-established correlation between biliary cysts and CCA has also been reported [15]. Bile duct cysts are a rare congenital disorder characterized by the development of cystic dilatation in any segment of the biliary tree, with an increased risk of both iCCA and eCCA, depending on the cysts’ distribution [16]. The overall lifetime incidence of CCA in this population with bile duct cysts is reported to be 6% to 30% [15], corresponding to a 20- to 30-fold increased risk compared with healthy individuals [4]. Caroli’s disease, defined by the presence of nonobstructive saccular or fusiform dilatation of larger intrahepatic bile ducts, represents the bile cyst phenotype most commonly complicated by CCA [16], with a 38-fold and a 97-fold higher risk for iCCA and eCCA, respectively, when compared to the general population [4].

Intrahepatic biliary lithiasis is strongly associated with iCCA [17], especially in East Asian cohorts and with larger gallstones [18], whereas the association between cholelithiasis and choledocholithiasis and CCA is more controversial. However, a recent meta-analysis [4] shows that these conditions, especially choledocholithiasis, are significantly associated with eCCA.

### 3.2. Chronic Liver Diseases

Chronic viral infections, especially hepatitis B and C virus (HBV and HCV, respectively), are risk factors for the development of CCA, with a stronger association with iCCA [4] and with a contribution to national CCA incidence rates that differs between Western countries and Asia, where HBV is endemic [19]. Notably, the increased risk of CCA in HBV and HCV patients is due to a direct carcinogenic effect of these viruses on target cells [19] and to chronic liver inflammation and the resulting hepatocellular and cholangiocellular proliferation, which increases the risk of malignant transformation [20]. Indeed, cirrhosis is a known risk factor for both hepatocarcinoma (HCC) and iCCA [4].

Similarly, a positive association between MAFLD and nonalcoholic steatohepatitis (NASH) and CCA has been suggested, especially in relation with iCAA, and even in the not-yet cirrhotic stage [20]. Finally, a positive but not significant association between hemochromatosis and iCCA was found in a population-based study (OR 2.1) [15].

### 3.3. Liver Fluke Infections

Parasitic infections, particularly *Opisthorchis Viverrini* and *Clonorchis Sinensis*, are the most common risk factor for the development of CCA in Asia [21]; for this reason, they are referred to as liver flukes and have been classified as group 1 biological carcinogens by the International Agency for Research on Cancer (IARC) [22]. Liver fluke infections are not carcinogenic per se, but the chronic inflammation (i.e., cholangitis) secondary to fluke infection/invasion within the bile ducts is associated with up to 5-fold increased risk of developing CCA in endemic areas [23]. Chronic infections and possible re-infections despite antihelminthic treatment lead to the development of CCA in up to 10% of people [24].

### 3.4. Genetic Factors

To date, there is very little information on inherited predisposing genetic risk factors for CCA [25]. Overall, polymorphisms of host genes encoding enzymes involved in xenobiotic detoxification, DNA repair, multidrug resistance, immune response, and folate metabolism have been linked to the development of CCA [26]. The available data largely derive from genome-wide association studies (GWAS) of cohorts of patients diagnosed with PSC and with an increased risk of CCA. However, the only detailed genomic CCA signature was found in cases caused by liver fluke infection: these subtypes of tumors have a higher overall mutation rate [27], with predominating mutations in SMAD4 and TP53 and ERBB2 amplifications [28]. KRAS mutations have been found in most CCA subtypes [29], and a significant association between TP53 mutation and HBV infection has also been observed [30]. Few studies have investigated the molecular distinction between the different anatomical localizations of the tumor: IDH, EPHA2, BAP1 mutations, and FGFR2 fusions are more common in iCCA, whereas extrahepatic tumors are mainly reported in PRKACA and PRKACB fusions [31], as well as ELF3 and ARID1B mutations [32]. These genomic differences may play a role not only in tumor location but also in therapeutic response: most of the ongoing phase III clinical trials on therapeutic strategies for iCCA are testing specific agents targeting IDH-mutated CCA (NCT02989857) and FGFR2 fusion-positive CCA (NCT03773302) [33,34].

Interestingly, however, the predominant genomic CCA alterations are associated with epigenetic processes [35]. Deregulated methylation patterns, histone modifications, and aberrant expression of non-coding RNAs have been shown to play important roles in cholangiocellular homeostasis and aberrant proliferation, and thus in the development and progression of CCA [36].

### 3.5. Metabolic Syndrome

Insulin-resistant patients have a higher overall cancer risk due to the mitogenic effect of insulin, which enhances a signaling pathway that activates the transcription of molecules involved in the cell cycle and proliferation [37]. Insulin-resistant patients have a higher overall cancer risk due to the mitogenic effect of insulin, which enhances a signaling pathway that activates the transcription of molecules involved in the cell cycle and proliferation [38,39]. However, data on the association between diabetes mellitus (DM) and CCA are not entirely clear. In the UK case-control study by Grainge et al., a significant association was found between DM and CCA and gallbladder cancer together (OR 1.39), but statistical significance was lost in the CCA subgroup analysis [39]. Similarly, in a Taiwanese population study by Chen et al. [40], a nonsignificant positive association was found between DM and CCA. Comparable results come from two other Asian case-control studies that focused specifically on iCCA [4,41]. In addition, a Chinese and, later, a British meta-analysis confirmed an increased independent risk of iCCA and eCCA in patients with type 2 DM (eCCA OR 1.5; iCCA OR 1.73, respectively) [4,42]. A possible protective role of metformin in the development of CCA has been suggested [37,43], even if further prospective studies are needed.

Regarding obesity, data on its association with the occurrence of CCA are conflicting and inconsistent [44]. Several mechanisms focusing on the role of leptin, adiponectin, insulin-like growth factor-1 (IGF-1), and inflammatory cytokines have been proposed to explain the effects of obesity on CCA [45]. Several studies and a meta-analysis reported a positive correlation between obesity and different types of CCA, especially for iCCA [43,46,47]. In contrast, several case-control studies [48] and a more recent meta-analysis by Clement et al. [4] found no significant association between obesity and CCA, reporting, on the contrary, that obesity was inversely associated with iCCA [49]. However, CCA patients with higher BMI certainly have worse outcomes [50].

Prospective studies are needed to better investigate obesity as a risk factor for CCA. Given the increasing prevalence of cardiovascular disease in Western countries and the fact that metabolic disorders are increasingly associated with primary liver cancer [51], a possible role of hypertension has been suggested. To date, most studies agree that there is a non-significant predisposition to CCA in patients with hypertension (iCCA OR 1.10, eCCA OR 1.21) [4].

### 3.6. Alcohol Consumption, Smoking, Races

Metabolism of alcohol by hepatic microsomes generates reactive oxygen and nitrogen species by lipid peroxidation, leading to the excretion of carcinogenic compounds (mainly formaldehyde) via bile, which causes DNA alterations.

Chronic alcohol abuse is identified as an independent risk factor for CCA, particularly for iCCA (iCCA OR 3.15, eCCA OR 1.75) [4], whereas moderate alcohol consumption may be associated with a lower risk of CCA (OR 0.82) [52]. This may be due to a reduction in cholesterol saturation, which in turn can help to reduce the formation of gallstones and protect against CCA [53]. Systematic investigation of the relationship between alcohol consumption at different doses and the occurrence of CCA certainly deserves future attention.

Tobacco smoking increases cancer risk worldwide through the direct and indirect carcinogenic effects of its toxic metabolites such as benzene and N-nitrosamines. Specifically, concerning CCA, these metabolites can either directly attack the epithelia of bile ducts or indirectly damage the DNA of gallbladder cells [54,55]. Although not statistically significant, most studies report a positive association between tobacco smoking and the occurrence of both types of CCA (iCCA OR 1.25 and eCCA OR 1.69, respectively) [4]. The few studies reporting an inverse association between tobacco smoking and CCA [44] may be biased by inadequate coverage of smoking habits in nationwide databases.

Very few studies have addressed epidemiologic differences in CCA among racial groups. Saha et al. examined trends in CCA incidence in the United States between 1973 and 2012 and reported that CCA incidence rates were 30% higher in Hispanics and Asians than in non-Hispanics and Caucasians [56]. Similarly, Patel et al. found that the US CCA incidence rate was higher among Asians and Pacific Islanders than Caucasians and African Americans between 2001 and 2015 [57]. A recent U.S. nationwide study by Baidoun et al. confirmed that Hispanics and Asian/Pacific Islanders are more likely to have CCA compared with Caucasians, whereas African American race is inversely associated with CCA [44]. These data may be biased by the coexistence of certified CCA risk factors in the populations studied.

### 3.7. Professional/Toxic Exposure

Recently, a link between asbestos exposure and CCA has been demonstrated in several case-control and cohort studies [58,59].These findings were confirmed in a population-based case-control study at the Nordic Occupational Cancer Cohort, where an increased risk for iCCA, but not eCCA, was observed from cumulative asbestos exposure [60].

Exposure to certain toxic substances has been consistently associated with the occurrence of CCA [61]. Thorotrast, a contrast agent containing thorium dioxide, a radioactive component with a long decay time (half-life of 400 years), was used in radiology from the early 1930s and was discontinued worldwide in the 1960s because it was strongly associated with delayed-onset liver cancer [62]. Patients exposed to thorotrast were found to have an up to 303-fold increased risk of CCA [63]. Table 1 shows the results of the available studies about established, emerging, and controversial risk factors for CCA.

## 4. Microbiota and Biliary Tract

The microbiota is a nonpathogenic microbial community living within the human body [64]. The interactions between the microbiota and the human host are important for host homeostasis [64,65,66]. Recently, many studies have been conducted to characterize the gut microbiota under physiological and pathological conditions. Physiologically, the intestinal barrier is the major line of defense preventing the translocation of bacteria from the intestinal lumen into the portal vein and then into the systemic circulation [67]. Alterations in the gut microbiota, defined as “dysbiosis,” lead to disruption of the intestinal barrier. Among different pathological conditions, this scenario may be associated also with some specific gastrointestinal diseases, such as IBD, chronic liver and biliary tract diseases such as PSC, and MAFLD [68,69,70]; in the latter case, a strong association between gut dysbiosis and liver metabolic disease has been explained by an increased hepatic ferroptosis. Ferroptosis is a novel form of programmed cell death caused by iron-dependent lipid peroxidation; it can be stimulated by intestinal bacterial invasion of the liver, and it has a key role in the pathological progression of MAFLD [70].

Generally, in the case of disruption of the intestinal barrier, the liver and bile ducts, which are physiologically sterile, can be exposed to the gut microbiota through the gut-liver axis, which is the major anatomical and physiological link between the gastrointestinal tract and the bile ducts and liver, passing through the systemic circulation [68]. Alterations in this axis contribute to the effect of the gut microbiota on the onset of various liver diseases, mainly cholangiopathies, through modified bile composition and altered biliary immunity. The biliary tract has an innate immune system that recognizes pathogen-associated molecular patterns through the action of Toll-like receptors (TLR) [71]. During gut bacterial invasion of the biliary tract, TLRs bind components of the bacterial cell wall including lipopolysaccharide (LPS) and trigger an inflammatory response in which cholangiocytes release a broad spectrum of pro-inflammatory cytokines, such as IL-1β, IL-8, IL-6, MCP-1, TNF-α, IFN-γ, and TGF-β, for pathogen elimination [72,73]. The aberrant inflammatory response is at the basis of different biliary tract diseases.

This is particularly the case for IBD patients, for whom intestinal barrier dysfunction and chronic inflammation are both triggers for the initiation and progression of tumor growth [74]. Alteration of the gut microbiota has been shown to influence the progression of PSC in IBD patients in some cases. Patients with ulcerative colitis (UC) who have PSC as an extraintestinal manifestation have an increased risk of CCA [75]. A recent study of liver samples showed that immune cell alteration, which is responsible for the occurrence of CCA, is higher in patients with PSC and active colitis than in patients with PSC and inactive colitis or PSC without colitis, suggesting a possible role of the microbiota in the association between colitis, PSC, and CCA etiopathogenesis in these patients [68]. In line with this hypothesis, these data may support the idea that antibiotics may represent a possible therapy for patients with colitis and PSC [76,77]. Indeed, treatment with oral vancomycin, which re-equilibrates the species of the gut microbiota, has been shown to improve liver enzymes and the natural history of biliary disease in patients with colitis and PSC. Vancomycin inhibits the growth of Gram-positive bacteria, including *Clostridia*, which are responsible for the conversion of primary to secondary bile acids that return to the liver through the entero-hepatic circulation. This indirect role of vancomycin in the biotransformation process may influence the composition of bile acids and underlie its effect on disease progression [69].

Recently, a role of the gut microbiota in cancer pathophysiology has also been observed, with both anti-tumorigenic and pro-tumorigenic effects. Among the species in the gut microbiota, *Propionibacterium*, for example, might induce apoptosis of colorectal cells through the production of short-chain fatty acids, while bacteria such as *Escherichia coli* and *Helicobacter* spp, which contain LPS, appear to be associated with the development of HCC [64]. With regard to the biliary tract, chronic activation of TLR and subsequent chronic inflammation of cholangiocytes induced by altered gut microbiota and dysfunctional gut-liver axis is associated with cholangiocytes proliferation and, in some cases, with their neoplastic transformation. The carcinogenetic role played by the chronic activation of TLR4 has already been linked to tumor proliferation and mononuclear antitumor activity inhibition in HCC, gastric carcinoma, and colorectal carcinoma [64].

## 5. Microbiota and CCA

### 5.1. Microbiota in CCA Pathogenesis

As previously mentioned, differences in gut microbiota composition have been observed in the development and progression of various cancers, due to the potentially carcinogenic effect of the inflammatory response resulting from bacterial translocation [77,78]. In this context, *Opisthorchis Viverrini* infection has been widely shown to be a risk factor for CCA. This infection is responsible for an alteration of the gut microbiota, and several clinical reports have observed an increase in *Helicobacter* spp in stool samples from these patients. In particular, overexpression of the *Helicobacter* genes CagA and CagE have been observed. The two gene-derived proteins migrate across the plasma membrane and cause phosphorylation of sarcoma family kinases, which may serve as transduction signals to promote fibrosis and inflammation of the bile ducts [79]. In vitro data have shown that CCA cells cultured with *Helicobacter* spp CagA+ express antiapoptotic factor bcl-2 more strongly and activate mitogen-activated protein kinase and nuclear factor-kappa B (NF-kB) signaling pathway, leading to further proliferation of bile duct cancer cells [80]. These data are consistent with a microbiota analysis of tumor samples from CCA patients performed by a specific polymerase chain reaction that showed an increase in three *Helicobacter* spp., including *H. Pylori*, *H. Bilis*, and *H. Hepaticus*. These samples also showed a strong inflammatory infiltrate and increased Ki67, indicating active cell mitosis [78,79,81].

Another hypothesis of a pathogenetic link between microbiota and CCA is related to myeloid-derived suppressor cells (MDSCs), which are immature myeloid cells with morphologic and phenotypic features of neutrophils (PMN-MDSC) and monocytes (M-MDSC), with different chemotaxis processes. Recently, the role of PMN-MDSC in the etiopathogenesis and progression of CCA has been described [82], as these immune cells facilitate tumor progression by suppressing cytotoxic T lymphocytes, enhancing angiogenesis, tumor invasion, and metastasis [82,83]. Moreover, as previously mentioned, TLR4 activation and high overexpression of the TLR4 gene are associated with CCA progression and worse disease outcomes, whereas lower TLR4 levels were associated with less tumor growth [29,68,84]. This observation is again consistent with a link between microbiota and CCA: altered microbiota and/or gut-barrier permeability alteration favor LPS translocation in the liver and biliary tract and activation of TLR4, determining chronic inflammation, tumor proliferation, and reducing mononuclear antitumor activity [64].

**Figure 1:**
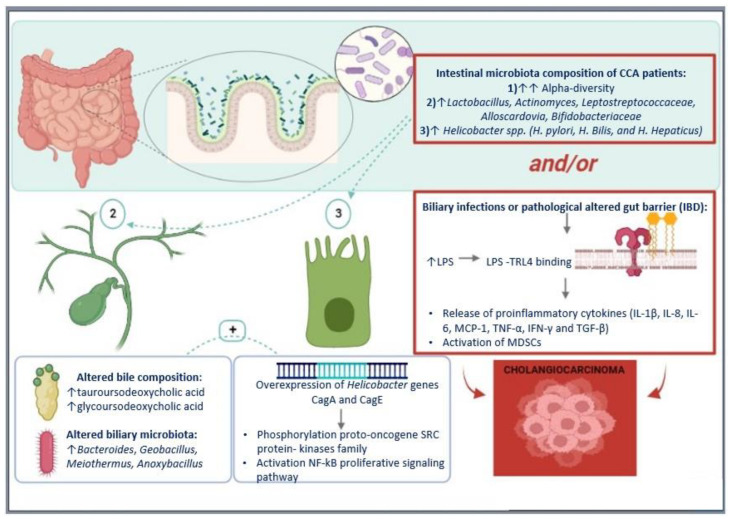
Microbiota alterations on CCA pathogenesis. Hypothesized role of the microbiota in cholangiocarcinoma and mechanisms of action.

To date, the most accurate data on the interaction between the biliary tract and gut microbiota in CCA pathogenesis come from studies in animal models showing that a disruption in the intestinal barrier leads to an accumulation of bacteria and LPS in the portal circulation and then in the liver and the biliary tree, resulting in hepatic and biliary recruitment of immunosuppressive cells [67], such as MDSCs. Furthermore, Zhang and colleagues [68] analyzed samples from mouse models of PSC, colitis, and CCA. Mice with PSC-like lesions after bile duct ligation showed major changes in the gut microbiota and signs of chronic colitis. This finding demonstrates that PSC may also cause an inflammatory response in the small intestine and impaired intestinal barrier function. In addition, a higher concentration of bacterial RNA was detected in the portal vein in these models, compared with controls. Mouse models with colitis induced by dextran sulfate also showed increased bacterial RNA concentration in the portal circulation and bacterial growth in the liver and lymph node tissue, in contrast to controls. These results indicate that there is a two-way continuous interaction between gut inflammation, gut microbiome, and hepatic host regulation, via gut-liver-axis communication. The same study [68] then compared liver samples from patients with PSC and active UC, PSC patients with inactive UC, and PSC patients without UC, evaluating the presence of PMN-MDS. The authors observed a higher concentration of PMN-MDSC in patients with PSC and active UC than in the other two groups [68]. These data indicate that disruption of the intestinal barrier of patients with active UC promotes the translocation of bacteria and LPS to the liver, resulting in activation of the inflammatory response and MDSC production. Figure 1 illustrates the mechanisms of action of microbiota alterations on CCA pathogenesis.

### 5.2. Microbiota in CCA Diagnosis

Based on the observed differences in the composition of the gut microbiota in malignancies, certain characteristics could be used as a noninvasive diagnostic tool. However, the most important factor in studying gut microbiota as a potential diagnostic tool is its highly dynamic variability and the consequent difficulty in defining a healthy control group. Indeed, daily stool samples from the same patient may show variations in bacterial composition, even if the composition remains stable in terms of percentages. Intestinal transit, genetic factors, lifestyle, eating habits, environmental factors, drug intake, and even bile composition contribute the most to intra- and interpersonal variations [81]. Despite this limitation, many studies to date provide increasing evidence on this topic. In this setting, Deng et al. [85] proposed a gut-microbiota-based signature based on eight genera (*Faecalibacterium*, *Klebsiella*, *Ruminococcus Gnavus group*, *Lactobacillus*, *Dorea*, *Veillonella*, *Burkholderia Caballeronia Paraburkholderia*, *Citrobacter*) that appears to have high accuracy in discriminating patients with CCA or HCC from healthy controls. The authors found that CCA patients had higher alpha-diversity (a measure of the diversity of the microbiota in a single sample) than HCC patients, while HCC patients had lower alpha-diversity compared with controls. Beta-diversity (a measure of the similarity or dissimilarity of two bacterial communities) was then assessed between the three groups: patients with CCA showed differences in microbiota profile compared to the other two groups, while HCC and control patients did not show significant differences, suggesting a similar bacterial distribution in the latter two groups. Based on these data, the authors finally extracted the eight genera for this diagnostic model [85].

In the multicenter study by Zhang et al. [78], the pattern of B-F-R genera (*Burkholderia-Caballeronia-Paraburkholderia*, *Faecalibacterium*, *and Ruminococcus-1*) was also suggested as a potential noninvasive biomarker to distinguish CCA patients from healthy controls. The authors investigated the differences in gut microbiota between CCA patients, patients with cholelithiasis, and healthy controls. CCA patients and healthy controls had a more species-rich and homogeneous microbiota than the cholelithiasis group, while there were differences in alpha-diversity between healthy controls and CCA patients. The authors finally identified the 20 most influential genera in patients with CCA and healthy controls and proposed this new diagnostic model that uses the presence and association of these three genera (B-F-R) in stool samples as diagnostic features for CCA [78].

Saab et al. [6] characterized the biliary microbiota of patients with eCCA and compared it with one of patients with cholelithiasis. To exclude confounding factors due to concomitant diseases, only patients without comorbidities were included for both groups. Patients with eCCA showed a significant difference in the composition of the biliary microbiota. In particular, the genera *Bacteroides*, *Geobacillus*, *Meiothermus*, *and Anoxybacillus* were significantly more abundant in the bile ducts of eCCA patients compared with controls. This suggests that eCCA is associated with bile duct dysbiosis, which allows these patients to be distinguished from controls with cholelithiasis.

Finally, another study by Jia and colleagues [86] characterized the gut microbiota of patients with iCCA. Patients with this type of cancer have greater amounts of *Lactobacillus*, *Actinomyces*, *Peptostreptococcaceae*, *Alloscardovia*, and *Bifidobacteriaceae* compared to patients with HCC or cirrhosis and healthy individuals. The authors found no significant differences in bile composition in the three populations studied, but they noted that the plasma/stool ratio (PSR) of tauroursodeoxycholic acid (TUDCA-PSR) and glycoursodeoxycholic acid (GUDCA-PSR) can be used to distinguish patients with CCA from other groups. In addition, TUDCA-PSR appears to have a strong positive correlation with the presence of *Lactobacillus* and *Alloscardovia* in the stool samples. Interestingly, in this study, it was observed that patients with CCA and vascular invasion (VI) had higher beta-diversity than patients with CCA without VI. However, the potential diagnostic significance of these associations needs further investigation [86]. In particular, it is not clear whether CCA induces changes in the homeostasis of bile acids via transporters and enzymes involved in bile acid metabolism, leading to alterations in the pool of their composition and allowing the predominance of certain bacteria, or whether the tumor induces dysbiosis due to altered environmental conditions (drug intake, dietary habits, concomitant infections, inflammatory status), which are then responsible for the different composition of bile acids.

### 5.3. Microbiota in CCA Prophylaxis

Emerging data from the characterization of the gut microbiota in CCA patients, which, if confirmed, could play a role in the diagnosis of this cancer, are encouraging and reinforce the idea that the microbiota could also be a therapeutic target. At present, several new molecules are under investigation for the prophylaxis of CCA in the areas of target therapy [87,88] and immunotherapy [87,89,90].

Since the alteration of the biliary immune system due to the influence of the gut microbiota plays an important role in the development of CCA, as shown by the available data in the literature, we can speculate that manipulation of the microbiota could soon have interesting therapeutic implications for this cancer.

## 6. Conclusions

The role of the microbiota in the microenvironment of CCA is an area of active research that is still not fully understood, although it may be associated with specific etiopathological mechanisms related to the carcinogenesis of CCA. However, there is evidence that the microbiota may play a role in the development and progression of CCA.

One perspective is that the microbiota may contribute to the development of CCA through its effects on the immune system and inflammation. Specifically, dysbiosis, or an imbalance in the microbiota, has been linked to a dysregulation of the intestinal barrier that may lead to an increased load of inflammation and immune dysfunction, which in turn may increase cancer risk. Moreover, the dysregulation of the intestinal barrier and the consequent bacterial contamination of the otherwise sterile bile ducts have been found to produce toxins or other substances that may promote the development of cancer. Both the biliary invasion by specific bacterial species and the PSR alteration of determined bile acids imply a chronic inflammation of the biliary tree, with a magnified pro-tumorigenic risk.

These aspects could be clinically important and may lead to considering the exciting implications of implementing both diagnostic and therapeutic strategies targeting the microbiota-immune system axis, especially in the subset of CCA.

From a diagnostic perspective, ongoing studies are searching for new biomarkers for early detection of CCA. In this scenario, future diagnostic perspectives should focus on the identification of a gut-microbiota-based signature of CCA, which must be reliable, reproducible, and the least influenced by environmental factors such as diet, lifestyle, and pharmacotherapy.

From a therapeutic perspective, targeting the microbiota could be a potential approach for the prevention or treatment of CCA. This could include the use of probiotics or other microbial therapies to alter the composition of the microbiota and potentially reduce the risk of CCA or slow its progression. Consequently, as far as prophylactic perspectives are concerned, microbiological and molecular research should focus on the development of technologies capable of effectively modifying the composition of the gut microbiota, whether this is fecal microbiota transplantation or the oral administration of antibiotics or probiotics, to specifically target the most commonly altered species in case of CCA, to restore the intestinal barrier, and thus to limit the consequent chronic biliary inflammation.

Ultimately, the intestinal microbiota could play a significant role in terms of response variation to standard pharmacological therapies for CCA. Future efforts should therefore also focus on the manipulation of the intestinal microbiota in order to optimize the response to these therapies. However, further research is needed to fully understand the role of the microbiota in CCA and to identify the most effective strategies to influence the microbiota to achieve therapeutic benefit.

## Figures and Tables

**Table 1:** Risk factors for CCA. Established, emerging, and controversial risk factors for CCA.

Factor	Type of Study	Calculated Risk
PSC	Population-based study	OR 22 for iCCA, OR 41 for eCCA
Bile duct cysts	Meta-analysis	OR 26.71 for iCCA, OR 34.94 for eCCA
Caroli’s disease	Population-based study	OR 38 for iCCA, OR 97 for eCCA
Hepatolithiasis	Population-based study	69% of the patients who underwent surgery for CCA had hepatolithiasis
Choledocholithiasis	Meta-analysis	OR 10.08 for iCCA, OR 18.58 for eCCA
Chronic hepatitis C	Meta-analysis	OR 4.28 for iCCA, OR 1.98 for eCCA
Chronic hepatitis B	Meta-analysis	OR 4.57 for iCCA, OR 2.11 for eCCA
Cirrhosis	Meta-analysis	OR 15.32 for iCCA, OR 3.82 for eCCA
MAFLD and NASH	Meta-analysis	OR 2.2 for iCCA, OR 1.5 for eCCA
Hemochromatosis	Population-based study	OR 2.1 for iCCA
Liver fluke (*Opisthorchis Viverrini*, *Clonorchis Sinensis*)	Meta-analysis	OR 5 iCCA > eCCA
Genetic factors	GWAS	IDH, EPHA2, BAP1 mutations, and FGFR2 fusions are more common in iCCA, and PRKACA and PRKACB fusions are more common in eCCA. Epigenetic hypermethylation of CpG early appears in CCA carcinogenesis.
Diabetes	Meta-analysis	OR 1.73 for iCCA, OR 1.5 for eCCA
Obesity	Meta-analysis	OR 1.14 for iCCA, OR 1.2 for eCCA
Hypertension	Meta-analysis	OR 1.10 for iCCA, OR 1.21 for eCCA
Alcohol consumption	Meta-analysis	OR 3.15 for iCCA, OR 1.75 for eCCA
Smoking	Meta-analysis	OR 1.25 for iCCA, OR 1.69 for eCCA
Races	Population-based study	Hispanics and Asian/Pacific Islanders are more likely to have CCA compared with Caucasians, and African American race is inversely associated with CCA
Asbestos	Case-control study	OR 4.8 for iCCA, OR 2.1 for eCCA
	Case-control study	OR 1.1–1.7 for iCCA, no association with eCCA
Thorotrast	Retrospective study	RR > 300

## Data Availability

Not applicable.
